# Azathioprine-Induced Pancreatitis in a Patient With Systemic Lupus Erythematosus

**DOI:** 10.7759/cureus.32697

**Published:** 2022-12-19

**Authors:** Haider Ghazanfar, Dongmin Shin, Ariyo Ihimoyan

**Affiliations:** 1 Gastroenterology, BronxCare Health System, New York City, USA; 2 Internal Medicine, BronxCare Health System, New York City, USA

**Keywords:** systemic lupus erythematosus, drug-induced pancreatitis, acute pancreatitis, azathioprine induced pancreatitis, azathioprine

## Abstract

Acute pancreatitis is an inflammatory disorder of the pancreas and a leading gastrointestinal cause of admission in the United States. The most common causes of acute pancreatitis are gallstones and alcohol. It is rarely caused by medications. Azathioprine-induced pancreatitis is rare and is more common in patients with Crohn’s Disease. In this case report, we present a rare case of azathioprine-induced pancreatitis in a 58-year-old African American patient with systemic lupus erythematosus.

## Introduction

Acute pancreatitis is an inflammatory disorder of the pancreas and a leading gastrointestinal cause of admission in the United States with an incidence ranging from 4.9 to 35 per 100,000 population [1,2]. The overall mortality of acute pancreatitis is approximately 5% but may vary depending on disease severity [3].

The most common causes of acute pancreatitis are gallstones and alcohol, accounting for about two-thirds of cases [4]. Other causes include various etiologies such as hyperlipidemia, hypercalcemia, drugs, viral infections, toxins, trauma, iatrogenic injury during surgery or endoscopic retrograde cholangiopancreatography (ERCP), vasculitis, ischemia, periampullary lesions, anatomical variants, and genetic mutations [5].

Although systemic lupus erythematosus (SLE) itself can cause pancreatitis through mechanisms such as vasculitis and thrombosis, less is known about azathioprine (AZA)-induced pancreatitis in SLE patients [6]. To the best of our knowledge, there have been no proven cases of AZA-induced pancreatitis after just one dose among those with SLE [7-9]. Here, we present our case of AZA-induced pancreatitis in a patient with SLE.

## Case presentation

Our patient is a 58-year-old African American female who came to the emergency department with a complaint of severe right upper abdominal pain for one day. The abdominal pain was sudden in onset, sharp in character, and radiated to her back. The pain worsened upon eating without clear relieving factors. Abdominal pain was associated with nausea and vomiting. The vomitus was non-bilious and non-bloody. She stated that the pain started one day after she took two tablets of azathioprine. This was the first time she had taken azathioprine. She denied any prior episodes of pancreatitis. Her past medical history was significant for hypertension, SLE (diagnosed 2 years ago), chronic kidney disease stage 4, and a history of stroke. Past surgical history was remarkable for cesarean section. She denied smoking, drinking alcohol, or using any recreational drugs. She denied taking any herbal medication or starting any new medication apart from azathioprine. Her regular medication included hydrochlorothiazide, ramipril, and prednisone.

On arrival at the emergency department, the patient had a blood pressure of 137/99 mmHg, heart rate of 98 beats per minute, respiratory rate of 18 breaths per minute, and a body temperature of 98.3°F. She appeared in mild distress due to pain. She had normal heart sounds and bilateral vesicular breathing. Abdominal examination was significant for right upper quadrant tenderness. Her initial laboratory result on presentation is shown in Table 1.

**Table 1:** Initial laboratory results MCV: mean corpuscular volume

Laboratory Parameter	Value	Reference Range
White blood cell count	4.8	4.8 - 10.8 k/uL
Hemoglobin	9.7	12.0 - 16.0 g/dL
Hematocrit	30.0	42.0 - 51.0%
MCV	95.8	80.0 - 96.0 fL
Platelet	164	150 - 400 k/uL
Sodium	136	135 - 145 mEq/L
Potassium	3.7	3.5 - 5.0 mEq/L
Bicarbonate	20	24 - 30 mEq/L
Chloride	102	98 - 108 mEq/L
Glucose	92	70 - 120 mg/dL
Blood urea nitrogen	23.0	8.0 - 26.0 mg/dL
Creatinine	1.9	0.5 - 1.5 mg/dL
Calcium	8.8	8.5 - 10.5 mg/dL
Albumin	3.7	3.4 - 4.8 g/dL
Total bilirubin	0.8	0.2 - 1.2 mg/dL
Direct bilirubin	0.2	0.0 - 0.3 mg/dL
Alkaline phosphatase	102	53 - 128 unit/L
Aspartate transaminase	30	9 - 48 unit/L
Alanine aminotransferase	12	5 - 40 unit/L
Total protein	6.3	6.0 - 8.5 g/dL
Ethanol level	< 10	≤ 10.0 mg/dL
Triglycerides	75	55 - 150 mg/dL
Lipase	912	≤ 61 U/L

**Figure 1:**
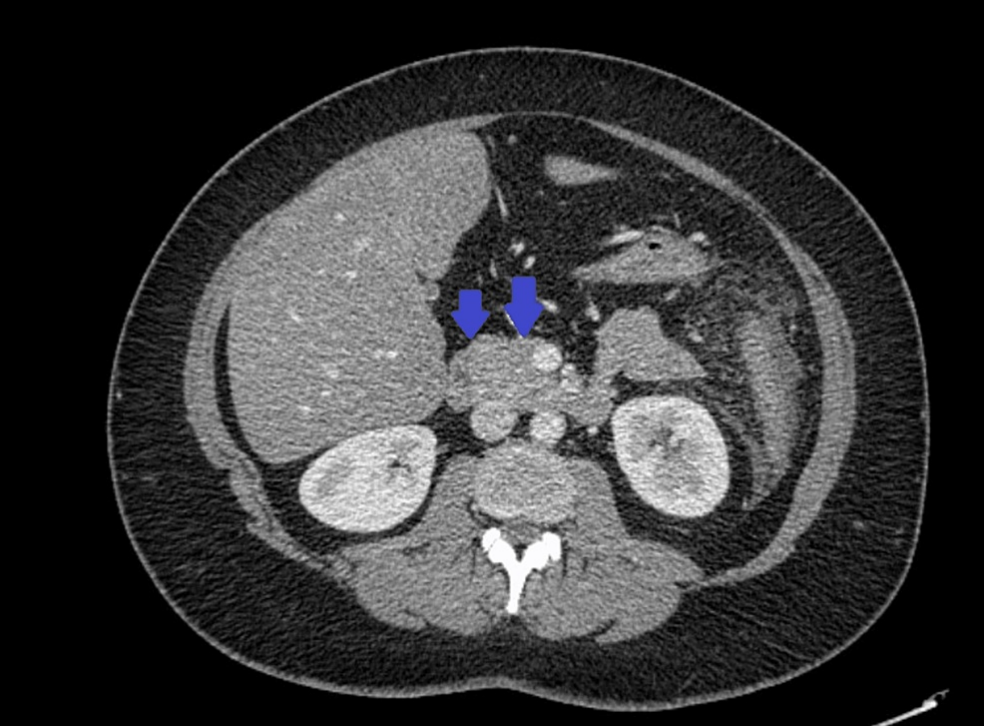
Computed tomography (CT) of the abdomen and pelvis with contrast showing acute pancreatitis (blue arrows)

Her chest X-ray was unremarkable. She underwent computed tomography (CT) of the abdomen and pelvis with contrast, which showed findings of acute pancreatitis. There was no gallstone or gallbladder wall thickening. There was no intrahepatic or extrahepatic biliary ductal dilation (Figure 1).

Ultrasound abdomen showed no gallstone with the common bile duct measuring 3 mm in diameter. The patient had no hepatomegaly or splenomegaly. Her anti-double stranded DNA (anti-dsDNA) level was normal, and she did not have any clinical signs or symptoms to suggest a lupus flare. She was started on aggressive intravenous hydration with Lactated Ringer’s solution. Her nausea, vomiting, and abdominal pain improved. She was started on a clear liquid diet and her diet was slowly advanced. Rheumatology was consulted and she was advised to stop taking azathioprine. AZA was held and the rest of her home medications were continued to optimize her medical comorbidities given that she never had prior episodes of pancreatitis despite being on those medications for a long time. Her clinical condition improved, and she was discharged with outpatient appointments.

Upon 3-month follow-up, the patient didn’t have any recurrence of pancreatitis despite being continued on her home medications except for azathioprine.

## Discussion

Up to 5% of acute pancreatitis are thought to be drug-induced, making it a rare cause of pancreatitis. Various drugs such as didanosine, valproic acid, thiazides, furosemide, pentamidine, chemotherapeutics, dexamethasone, prednisolone, estrogen, opiates, mesalamine/sulfasalazine, azathioprine, tetracycline, metronidazole, isoniazid, and rifampin have been implicated in drug-induced pancreatitis (DIP) [10]. These drugs are further classified based on their weight of evidence as the cause of pancreatitis [5,11].

**Figure 2:**
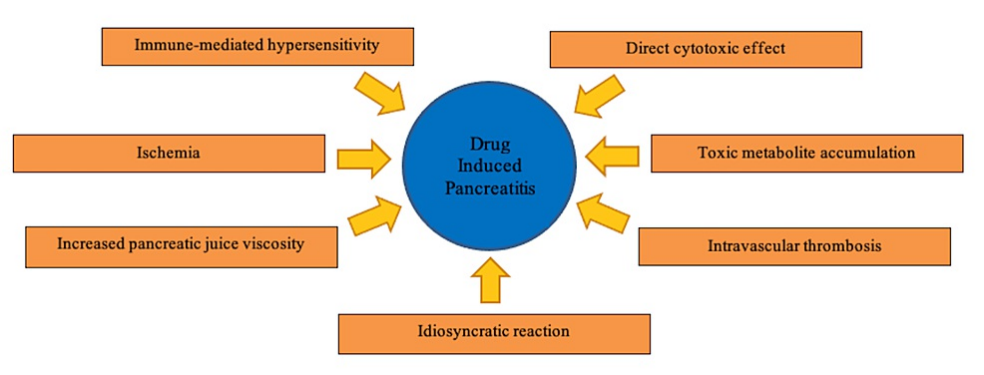
Mechanisms of drug-induced pancreatitis Image Credit: Haider Ghazanfar

Certain drugs can cause pancreatitis through a wide variety of mechanisms such as immune-mediated hypersensitivity reaction, direct cytotoxic effects, toxic metabolite accumulation, ischemia, intravascular thrombosis, increased pancreatic juice viscosity, and idiosyncratic reaction [5,12]. This has been presented in Figure 2.

AZA is a known medication that can cause idiosyncratic DIP [13]. Studies of inflammatory bowel disease patients on AZA revealed an incidence of AZA-induced pancreatitis of 7.5%, with a mean time of 25 days until developing pancreatitis from drug initiation, and a mean dose of 88 mg. All patients had a mild course of disease that resolved after discontinuing the medication. Smoking, concomitant budesonide use, and a single daily dose of AZA were found to be risk factors for AZA-induced pancreatitis. Those who smoked more than 3 weekly packs were more likely to get AZA-induced pancreatitis compared to patients who smoked less than or equal to 3 weekly packs [14,15]. Other studies have also suggested that certain human leukocyte antigen (HLA) types are associated with an increased risk of AZA-induced pancreatitis [16,17]. Crohn’s disease is the most commonly encountered condition among patients with AZA-induced pancreatitis, compared to other conditions [7]. Risk factors for developing AZA-induced pancreatitis are shown in Table 2.

**Table 2:** Risk factors for azathioprine-induced pancreatitis [7,14-17] HLA: human leukocyte antigen

Risk Factors
Smoking
Concomitant budesonide use
Single daily dose of azathioprine
Genetic variants in the HLA gene region
Crohn’s Disease

Diagnosing DIP can be challenging. In order to definitively prove DIP, other possible causes of pancreatitis should first be ruled out, pancreatitis should develop after exposure to the culprit drug and resolve upon discontinuation of the drug, and pancreatitis should recur upon re-exposure to the drug. However, most reported cases lack evidence from drug rechallenge, which is a crucial part in proving the causal relationship. The high incidence of concurrent illness known to induce acute pancreatitis also obscures the causal relationship with the drug [10,18].

One study showed that DIP may be overlooked, and its prevalence may be higher than previously recognized. Since the discontinuation of the culprit drug is key to DIP management, it is important for physicians to be aware of the various drugs that can cause DIP and consider it as the diagnosis in those with otherwise unexplained acute pancreatitis [19]. Most patients with DIP have a mild benign disease course with a good prognosis [13,20].

## Conclusions

Our case highlights the importance of having a high clinical suspicion of azathioprine-induced pancreatitis in a patient with SLE. AZA should be held at the time of admission in patients with high clinical suspicion of DIP while waiting for a workup to rule out other common causes of pancreatitis. Our patient had an uncomplicated hospital course with discontinuation of AZA and conservative management.
